# Territoriality ensures paternity in a solitary carnivore mammal

**DOI:** 10.1038/s41598-017-04820-4

**Published:** 2017-07-03

**Authors:** Francisco Palomares, María Lucena-Pérez, José Vicente López-Bao, José Antonio Godoy

**Affiliations:** 1 Department of Conservation Biology, Estación Biológica de Doñana (CSIC), Avda. Américo Vespucio s/n, Isla de la Cartuja, 41092 Sevilla, Spain; 2 Department of Integrative Ecology, Estación Biológica de Doñana (CSIC), Avda. Américo Vespucio s/n, Isla de la Cartuja, 41092 Sevilla, Spain; 3 Research Unit of Biodiversity (UO/CSIC/PA), Oviedo University, 33600 Mieres, Spain

## Abstract

In solitary carnivorous mammals, territoriality is assumed to benefit male fitness by ensuring the exclusivity of matings within territories via mate guarding and female defence. However, this hypothesis remains empirically untested. Here, we examined this hypothesis for solitary territorial carnivores using the Iberian lynx *(Lynx pardinus)* as a case study. We expected that territorial males sire all litters born within their territories, translating into the absence of multi-paternity cases within the same litter. We analysed parentage in 43 kittens, belonging to 20 different litters. For 42 kittens, a father could be assigned using microsatellites and always coincided with the individual holding the territory. For 16 kittens from 10 litters for which we also had information on SNPs, paternity assignments coincided with microsatellites, except for a litter (two kittens) from the same litter for which a different male was assigned, but the territorial male could not be excluded. Our results indicated that multi-paternity in the Iberian lynx must be a rare event, and that territorial males sire all litters born from the females with which they share territories. We propose that both the low number of mature individuals in the lynx population and the fact that female oestrus is induced by male presence may explain results.

## Introduction

The spacing pattern in mammal populations, such as the defence of exclusive animal home ranges (i.e., territoriality), is the result of the tactics chosen by individuals to maximize their fitness^1–3^. In the case of females, food acquisition and defence of offspring have been identified as important drivers of their spatio-temporal distribution^3–5^. Consequently, it is expected that the distribution of females will determine the spacing patterns of males, at least during the mating season^1, 2, 6^. Male mammals show a diverse array of mating bonds, including obligate monogamy, unimale and group polygyny and promiscuity^7^. These are associated with a wide variety of different forms of mate guarding, including for instance, the defence of female groups or receptive females.

In solitary carnivorous mammals, where males are not involved in parental care, male reproductive success is largely limited by access to females and their defence from other males, rather than by acquisition of nutrients^1^. This results in a strong territorial spacing system directed at monopolizing access to females^1, 8, 9^. Thus, territorial males are expected, theoretically, to expel intruders in order to maximize their fitness. Under this premise, it can be hypothesized that the energetic cost of territoriality for resident males is compensated for by the monopolization of mating opportunities with females within male territories^10^. As multi-male mating by females is common in mammals from different families with varying life histories, mate guarding may have evolved in response to the threat of infanticide and the subsequent tendency of females to mate multiply^5^.

In this study, we comprehensively examined the mate guarding and female defence hypothesis for solitary territorial carnivores. We used the Iberian lynx (*Lynx pardinus*), a solitary, resident, and territorial carnivore^11^, as a model species. We combined long-term datasets of genetic (to evaluate genetic paternity of kittens) and field data (radio-tracking and camera-trapping data) from the Doñana lynx population (SW Iberia). Over the last few decades, lynx have been extensively studied within the Doñana area^12–14^. The Iberian lynx exhibit a territorial spacing pattern both in males and females, with male territories overlapping with either one or several females, depending on female territory size and male availability^4, 11, 12^. Home range size and territoriality in females have been related to food availability and possibly to the defence of offspring from infanticide^4, 15, 16^. However, for males, mate guarding and female defence have often been assumed, but not empirically tested^11, 12^. Under the mate guarding and female defence hypothesis, we predict that territorial males should sire all litters born from females overlapping with their territories. Consequently, it should be expected that multi-paternity events do not occur, or are extremely rare.

## Results

Over the study period (1995–2008), we compiled information for a total of 43 kittens (all successfully genotyped by microsatellites and 16 by SNPs), grouped into 19 litters (17 from Coto del Rey and 3 from La Vera), involving 10 different females and 11 different males (Table 1). In 17 out of 20 litters, we knew that in the nucleus there were between 1 and 3 adult males besides the male overlapping with the focal female territory.

**Table 1:** Litters from Iberian lynx from Doñana National Park where paternity assignments were performed.

Litters					Microsatellites		SNPs	
Nucleus^1^	Mother	Year	Spatial father^2^	Other known males in the nucleus	Number genotyped kittens (total kittens in the litter)	Genetic father	Number genotyped kittens	Genetic father
CR	Nuria	1995	Borja (RT)	Maki, ungenotyped	2 (3)	Borja	0	—
CR	Gloria	1996	Barro (RT)	Borja, Maki, ungenotyped	2 (?)	Barro	2	Barro
CR	Gloria	1997	Barro (RT)	Maki, ungenotyped	3 (3)	Barro	1	Barro
CR	Nuria	1997	Barro (RT)	Maki, ungenotyped	1 (3)	Barro	1	Barro
CR	Iguazu	2000	Barro (CT)	Uda, Oscar	1 (3)	NA^3,4^	1	NA^4^
CR	Roja	2000	Barro (CT)	Uda, Oscar	2 (4)	Barro	2	Barro
CR	Escarlata	2000	Barro (CT)	Uda, Oscar	1 (2)	Barro	1	Barro
CR	Viciosa	2002	Oscar (CT)	Barro, Uda	3 (3)	Oscar	2	Uda
CR	Wari	2004	Uda (CT)	Oscar	2 (2)	Uda	1	Uda
VE	Jabata II	2006	Pavon (RT)	—	2 (2)	Pavon	0	—
CR	Wari	2006	Roman (RT)	Arrayan, Nati II	2 (2)	Roman	1	Roman
CR	Rayuela	2006	Nati II (RT)	Roman, Arrayan	2 (2)	Nati II	0	—
CR	Viciosa	2006	Arrayan (RT)	Roman, Nati II	2 (2)	Arrayan	0	—
CR	Viciosa	2007	Arrayan (RT)	Roman, Nati II	3 (3)	Arrayan	0	—
CR	Wari	2007	Roman (RT)	Arrayan, Nati II	2 (3)	Roman	0	—
CR	Rayuela	2007	Nati II (RT)	Roman, Arrayan	2 (2)	Nati II	0	—
CR	Viciosa	2008	Baya (RT)	—	3 (3)	Baya	2	Baya
VE	Bonares	2008	Clavo (RT)	Boliche	2 (2)	Clavo	0	—
CR	Wari	2008	Baya (RT)	—	4 (4)	Baya	3	Baya
VE	Jabata II	2008	Boliche (RT)	Clavo	2 (2)	Boliche	0	—

Information on the nucleus in which the litter was located, the mother of the litter, the supposed father according to spatial data, the number of known adult males in the nucleus, the number of genotyped kittens by any method, total number of kittens in the litter (between brackets; ?=unknown) and paternity assignments using microsatellites and SNPs information. Paternity assignment for microsatellites was performed for two scenarios (not assuming and assuming monogamy of females). Detailed probabilities for all cases are shown in Supplementary Table S1. ^1^CR = Coto del Rey, VE = Vera. ^2^Between brackets methods of spatial father determination: RT = radio-tracking; CT = camera-trapping. ^3^NA = not assigned. ^4^The potential father was categorically excluded as a father of the kitten by both microsatellite and SNP genotype data.

For none of the 17 litters for which we obtained microsatellite genotypes of 2 to 4 kittens, the data indicated multiple paternities when we did not assume monogamy of the female (Table 1). Paternity assignments were thus identical in both scenarios, but probabilities were higher when assuming monogamy (Supplementary Table S1). Similarly, for none of the five litters for which we genotyped 2 to 3 kittens using SNPs (number total of kittens = 16), data suggested multiple paternity (Supplementary Table S1).

Out of the 43 kittens successfully genotyped, in 40 cases (93%) we identified the male suggested by field data as the most likely father using microsatellites (Table 1). Paternity probability assignment was 1, >0.9 and <0.5 in 26.2%, 45.2%, and 4.8% of cases, respectively, for the scenario that did not assume monogamy in females (Supplementary Table S1). These probabilities changed to 57.1%, 26.2% and 0%, respectively, for the scenario that assumed monogamy in females (Supplementary Table S1). For the kittens where we also had information on SNPs, paternity assignments coincided with those of microsatellites in all cases except for two kittens from the same litter (Viciosa 2002), in which there was a discrepancy (Table 1). The assignment was ambiguous with microsatellites, as the probability that Oscar, the territorial male, had sired the kittens was only slightly higher than that of Uda (P = 0.519–0.620 and 0.346–0.478 for Oscar and Uda, respectively), and both had zero mismatches with the two kitten-mother duets. This was due to both candidates having very similar genotypes and an estimated relatedness of 0.9 (Supplementary Table 2). Only Uda was genotyped for SNPs and he presented zero mismatches with both offspring-mother duets (out of 1,406 loci compared). However, since Oscar could not be genotyped with SNPs we considered this an unresolved ambiguity. In a single litter (Iguazú 2000) we could exclude the territorial male as the father of the single genotyped kitten: no male was assigned, and the potential father (Barro) accumulated 4/35 microsatellite and 23/1397 SNP mismatches with the genotyped kitten in this litter.

## Discussion

Our results indicate that multiple paternity in the Iberian lynx must be an extremely rare event, if it occurs at all, and that territorial males sired almost all litters born from females overlapping with their territories. There was only one litter with some discrepancy in paternity assignment: according to microsatellites the overlapping male sired the litter, whereas SNPs assigned another male of the same nucleus as the father. It is not rare for Iberian lynx males to perform excursions into neighbouring territories during the mating period, which may allow for paternity theft or multiple male mating by females^11, 17^. However, the microsatellite genotypes of these two conflicting males were totally compatible with kittens and females, in addition to be highly similar between them (Supplementary Table S2). This makes us to be cautious and do not discard a possible genotyping error or confusion between samples. So we cannot rule out that the territorial male sired the litter rather than the other male. On the other hand, there was another kitten with no father assigned (the only one genotyped in the litter) despite the fact that its potential father according to field data, and two other males, were genotyped in the same area and included as candidate parents in the analyses. Furthermore, in this case, the territorial male was categorically excluded with high confidence as a parent of the only kitten genotyped by SNP data. It is worth noting that the excluded territorial male did successfully breed that same year with two other females occupying territories adjacent to this female. Thus, this male did successfully defend two females, but data suggest that he failed in the defence of a third one.

Our results confirm the mate guarding and female defence hypothesis in the case of the Iberian lynx. This hypothesis predicts that territorial males, or socially dominant males in species that live in groups, should sire all litters born within their territories and, therefore, multiple paternity within the same litter or litter paternities from other males should not be expected^1, 8, 9^. Territorial or socially dominant males would be expected to monopolize mating opportunities with females, maximizing their fitness^10^. Male mammals associating with females either socially or spatially would try to guard, or conceal, those females from other males^8, 9^. Despite the theoretically accepted importance of the behaviour of mate guarding and female defence by males, in general there is little empirical information on the effectiveness of this behaviour in assuring reproduction for most species.

Recent studies on genetic parentage and relatedness have showed that male mate guarding is somewhat ineffective in gregarious species, or species that live in groups comprising a dominant mated pair and theoretically one or more related, but reproductively suppressed, subordinates (i.e., where social monogamy is expected). For example, non-genetic monogamy and cases of mixed paternities have been reported in populations of canids (red fox, *Vulpes Vulpes* ^18^; African wild dogs, *Lycaon pictus* ^19^; Island foxes, *Urocyon littoralis* ^20^), herpestids (dwarf mongooses, *Helogale parvula* ^21^), procyonids (white-nosed coati, *Nasua narica* ^22^; southern Illinois raccoons, *Procyon lotor* ^23^), mustelids (Eurasian badgers, *Meles meles* ^24^) and felids (African lions, *Panthera leo* ^25^). However, to our knowledge, our study and that of Hedmark *et al*.^26^ with wolverines (*Gulo gulo*), are the only studies in mammalian carnivores where empirical support for the mate guarding and female defence hypothesis has been provided. These two species share an intra-sexual territorial spacing system, and a polygamous social system with males, able to overlap territories and reproduce with several females^11, 12, 26, 27^. Empirical genetic confirmation of expected paternity between males and females sharing space in other mammal species is also scarce (but see refs 28–30, who reported genetic and behavioural monogamy in the old field mouse, *Peromiscus polionotus*, the California mouse *P. californicus*, and the dwarf antelope, Kirk’s dik-dik, *Madoqua kirkii*, respectively).

On the contrary, extra-pair copulation and multiple paternity is known to occur in several mammal species where territorial or social dominance is expected, including carnivores from eight families, among them felids^6^. The most convincing explanation is that multi-male mating confuses paternity, thereby deterring infanticide by males^6^. Mate guarding and, perhaps even in some cases, behavioural monogamy, may have evolved in response to the threat of infanticide and the subsequent tendency of females to mate multiply. Infanticide has been reported in one case, and is suspected in another case in Iberian lynx^16^, and in fact multi-male mating is suspected to occur occasionally^17^. Nevertheless, although we can not discard the Iberian lynx females are able to mate in occasions with several males in the same breeding season, our results indicated that they can be considered as genetic monogamous, since we did not detect any case of multiple parternity. The absence of multiple paternity or that males from neighbouring territories are not able to sire litters, has been explained by the fact that males of these species show high levels of litter care^31, 32^. In the *Peromiscus* species mentioned earlier, males generally show a substantial amount of parental care^31^, similar to that in numerous monogamous birds, where high levels of male care are often associated with a high degree of paternity certainty^32^. However, this explanation does not apply to the results found for Iberian lynx since males do not participate in the care of young^11^ (also see ref. 33, for the case of dik-diks).

In the case of the Iberian lynx population studied here, the absence of multiple paternity may be related to two non-exclusive factors. First, the Doñana Iberian lynx population is critically threatened by extinction, and many optimal territories used to be empty or occupied by immature individuals (see refs 34, 35 for details). Therefore, a low number of mature individuals could favour mate guarding and female defence by males. Similarly, Hedmark *et al*.^26^ also proposed that the low density might explain the social monogamy found in the wolverine population they studied. In these situations, males can concentrate on female defence during the oestrus and ovulation phase. Second, the oestrus phase in both species is induced by male presence^36, 37^ and, at least in the Iberian lynx, it usually takes only 2–3 days (A. Vargas, pers. comm.). In this situation, female guarding from neighbouring males may be a relatively easy task for males.

## Methods

### Lynx data, sample collection and genotyping

We used data gathered from 1995 to 2008 from two lynx nuclei within Doñana National Park (37°10′ N, 6°23′ W), namely Coto del Rey and La Vera (Table 1; see refs 4, 11, 12, 38; for detailed descriptions of the Doñana area and its lynx population). These two nuclei are separated by the marsh and there is practically no contact between lynx living in each nucleus^11, 12^. Data on litter size and their spatial position, maternity of the litters, and spatial information of the mother and the potential father of kittens were obtained through capture, radio-tracking and camera-trapping activities (following the procedures described in refs 11–13, 39). The exact birth dates of all animals for which paternity analyses were undertaken were known because mothers were previously tagged with VHF collars, except in one case where the female lynx was known from their unique pattern of spots^13^ and collared later. All methods of capture, handling and collaring were specifically approved by the Regional Government of Andalusia and Doñana National Park, and complied with the norms of the Spanish Animal Protection Regulation RD1201/2005.

Males were considered potential fathers of the litter when, during the mating period (December-April), simultaneous radio-tracking data of males and females was available, and the female territory overlapped with the male territory. In Coto del Rey nucleus adult female lynx territories usually overlapped with only one adult male territorry^12^, however in La Vera it was more common an adult female could overlap her territory with more than one adult male (until 3 different males recorded), although in most cases with one male overlap >50% of her territory^11^. In these cases, the male overlapping more than 50% of female territory was considered the potential father of the litter. We also considered a male as a potential father in cases where only the female was radio-tracked, but there was at least one photograph of the male during the mating period within the female territory taken by a camera station situated ≤1 km from where the kittens were born. Accumulating evidence from dozens of adult lynx collared in this area indicates that both males and females maintain territories for years, and are easily observed and photographed when holding a territory^4, 11, 12^. Ranges of home range sizes estimated using the minimum convex polygon with 95% of locations for females and males in the two study nuclei of Doñana were 3.9–6.6 km^2^ and 4.8–18.3 km^2^ in Coto del Rey, and 7.5–24.6 km^2^ and 8.5–25.0 km^2^ in La Vera, respectively^11, 12^. For 75% of litters spatial relationships between female and the supposed father of kittens was known from radio-tracking data, and for the remainder 25% from camera-trapping data (Table 1).

We collected blood, tissue and faeces for genetic analyses from captured or necropsied lynx and from museum specimens in the collection of Doñana Biological Station (see 14 for details). All lynx for which paternity was analysed were tagged with transponders when they were 1–3 weeks old^16, 39^. Thus, these animals could be identified again when they were captured for collaring or when they were found dead. From all lynx samples, DNA was extracted and used to genotype up to 36 microsatellite loci (details on genotyping can be found in Casas-Marce *et al*.^14^). Some of these same lynx were additionally genotyped for 1,468 autosomal SNPs^40^.

### Parentage analyses

We inferred parental-offspring relationships using Colony 2 for Mac^41^. Colony 2 uses a maximum likelihood approach to calculate parentage for a given year/cohort and allows the inclusion of a priori information, such as allelic frequencies of the population, mating system, genotype error rates and known parent-offspring relationships. We estimated the allelic frequencies from the animals born during the study period and assumed an error rate of 0.01 for microsatellites and 0.0002 for SNPs (both considered as a conservative estimations of the real error rate). As previous analysis showed a limited power for microsatellites to estimate paternities correctly if field information was disregarded (Lucena-Pérez *et al*. unpubl. data), we included the relationships mother-kittens, which was known for all the litter analysed. Thus, reliability of the analysis was high as the assignments of kittens to the true mothers were 1 in 39 cases, and between 0.954 and 0.999 in the four remaining ones (Supplementary Table S1). Besides, for every cohort, we set as candidate fathers all males between 2 and 12-years-old, both ages included, known to be in every lynx nuclei according to the criteria mentioned above. Candidate fathers were in some instances related to the known mother or between them (Supplementary Table S2). The percentage of the potential fathers genotyped and included in the analysis was estimated as the ratio between the number of males genotyped in a nucleus and the known number of males in the nucleus plus 1 (to account for unlikely unregistered visits of males from different areas). We performed the analyses under two scenarios: females are monogamous, and females are polygamous. In both scenarios, males were assumed to be polygamous as they can mate with more than one female in a breeding season. For SNPs, we did not include any prior known relationship between the individuals.

All the input data for Colony 2 was written using a Python script that extracted the information from a SQL database where we stored all the relevant demographic and genotypic information (https://github.com/mlucenaperez/paternity_analysis). More details on paternity analyses are available in M. Lucena-Pérez *et al*. (unpubl. data).

### Data Availability

The datasets generated during and/or analysed during the current study are available from the corresponding author on reasonable request.

## Electronic supplementary material

Supplementary information
