# Supplementary material for: Territoriality ensures paternity in a solitary carnivore mammal
Source: Sci Rep. 2017 Jul 3;7:4494. doi: 10.1038/s41598-017-04820-4 (PMC5495821; doi:10.1038/s41598-017-04820-4)
Supplement: Supplementary file 1 — Supplementary information [file 41598_2017_4820_MOESM1_ESM.pdf]

Territoriality ensures paternity in a solitary carnivore mammal

Francisco Palomares, María Lucena-Pérez, José Vicente López-Bao and José Antonio Godoy

Supplementary Table S1. Parentage assignments of Iberian lynx litters from Doñana National Park. Information on the litters (nucleus of Doñana in which the litter was located, the year, the mother, the supposed father according to spatial data, and the known adult males living in the nucleus), the identity of kittens genotyped, and the mother and father assignments and their probabilities are shown. Maternity and paternity assignments with microsatellites were performed under two scenarios, not assuming and assuming monogamy of females, and with SNPs not assuming monogamy of females. Both probabilities are reported (P_{nm} and P_{m}, respectively) for microsatellites; mother assignment probabilities were identical in both scenarios. In bold the IDs of females, males and kittens which were also genotyped by SNPs (all individuals were genotyped by microsatellites). ; ni: male not included among the inferred fathers; na: not applicable (offspring or candidate father not genotyped for SNPs); ^{s} maternity assignment confirmed also by SNPs.

Litter field information	Genetic parentage assignment

Nucleus	Year	Mother	Spatial father	Other known males in the nucleus	Id genotyped offspring	Maternal Microsatellite probabilities	Id candidate fathers	Paternal Microsatellite probabilities P_{nm}, P_m	Paternal SNPs probabilities
CR	1995	**Nuria**	**Borja**	**Maki**, ungenotyped	Hollywood	1	**Borja**	0.989, 1	na
							**Maki**	ni, ni	na
					Roja	1	**Borja**	0.997, 1	na
							**Maki**	ni, ni	na
CR	1996	Gloria	**Barro**	**Borja**, **Maki**, ungenotyped	**Uda**	1	**Barro**	0.997, 1	1
							**Borja**	ni, ni	ni
							**Maki**	ni, ni	ni
					**Yolanda**	1	**Barro**	1, 1	1
							**Borja**	ni, ni	ni
							**Maki**	ni, ni	ni
CR	1997	Gloria	**Barro**	**Maki**, ungenotyped	Elsa	1	**Barro**	0.978, 0.995	na
							**Maki**	ni, ni	na
					Iguazu	1	**Barro**	0.301, 0.995	na
							**Maki**	ni, ni	na
					**Lucia**	1	**Barro**	0.956, 0.995	1
							**Maki**	ni, ni	ni
CR	1997	**Nuria**	**Barro**	**Maki**, ungenotyped	**Javitxu**	1^S	**Barro**	0.999, 1	1
							**Maki**	ni, ni	ni
CR	2000	Iguazu	**Barro**	Uda, Oscar	**Viciosa**	1	**Barro**	ni, ni	ni

							Uda	ni, ni	ni
							Oscar	ni, ni	na
CR	2000	Roja	**Barro**	Uda, Oscar	**Guinness**	1	**Barro**	0.746, 1	1
							Uda	0.143, ni	ni
							Oscar	0.106, ni	na
					**Zoe**	1	**Barro**	1, 1	1
							Uda	ni, ni	ni
							Oscar	ni, ni	na
CR	2000	Escarlata	**Barro**	Uda, Oscar	**Rada**	0.999	**Barro**	0.997, 0.997	1
							Uda	ni, ni	ni
							Oscar	ni, ni	na
CR	2002	**Viciosa**	Oscar	**Barro**, Uda	**Aura**	1^{S}	Oscar	0.558, 0.646	na
							Uda	0.346, 0.354	1
							**Barro**	0.09, ni	ni
					**Telma**	1^{S}	Oscar	0.620, 0.646	na
							Uda	0.379, 0.354	1
							**Barro**	ni, ni	ni
					Viana	1	Oscar	0.519, 0.646	na
							Uda	0.478, 0.354	na
							**Barro**	0.002, ni	na
CR	2004	Wari	Uda	Oscar	**Almoradou**x	1	Uda	0.632, 0.732	1
							Oscar	0.365, 0.268	na
					Angustias	1	Uda	0.624, 0.732	na
							Oscar	0.354, 0.268	na
VE	2006	**Jabata II**	**Pavon**	-	Cachuela	1	**Pavon**	0.692, 0.908	na

					Clavo	1	**Pavon**	0.788, 0.908	na
CR	2006	Wari	**Roman**	Arrayan, Nati II	Calendula	1	**Roman**	1, 1	na
							Arrayan	ni, ni	na
							Nati II	ni, ni	na
					**Centaurea**	1	**Roman**	0.997, 1	1
							Arrayan	ni, ni	na
							Nati II	ni, ni	na
CR	2006	Rayuela	Nati II	**Roman**, Arrayan	Cicuta	1	Nati II	0.989, 1	na
							**Roman**	ni, ni	na
							Arrayan	ni, ni	na
					Coca	1	Nati II	0.972, 1	na
							**Roman**	ni, ni	na
							Arrayan	ni, ni	na
CR	2006	**Viciosa**	Arrayan	**Roman**, Nati II	Canyamo	1	Arrayan	0.987, 0.994	na
							Nati II	0.003, ni	na
							**Roman**	ni, ni	na
					Carrizo	1	Arrayan	0.989, 0.994	na
							Nati II	ni, ni	na
							**Roman**	ni, ni	na
CR	2007	**Viciosa**	Arrayan	**Roman**, Nati II	Dehesa	0.962	Arrayan	0.967, 0.997	na
							**Roman**	ni, ni	na
							Nati II	ni, ni	na
					Delibes	0.996	Arrayan	0.998, 0.999	na

							**Roman**	0.001, 0.001	na
							Nati II	ni, ni	na
					Lcm166	0.954	Arrayan	0.997, 0.999	na
							**Roman**	ni, ni	na
							Nati II	ni, ni	na
CR	2007	Wari	**Roman**	Arrayan, Nati II	Daroeira	1	**Roman**	0.998, 1	na
							Arrayan	ni, ni	na
							Nati II	ni, ni	na
					Durillo	1	**Roman**	0.993, 1	na
							Arrayan	0.004, ni	na
							Nati II	ni, ni	na
CR	2007	Rayuela	Nati II	**Roman**, Arrayan	Dalia	1	Nati II	0.506, 0.503	na
							**Roman**	ni, ni	na
							Arrayan	ni, ni	na
					Daphne	1	Nati II	0.499, 0.503	na
							**Roman**	ni, ni	na
							Arrayan	ni, ni	na
CR	2008	**Viciosa**	**Baya**	-	Epsilon	1	**Baya**	1, 1	na
					**Esencia**	1^{S}	**Baya**	1, 1	1
					**Estepa**	1^{S}	**Baya**	1, 1	1
VE	2008	Bonares	Clavo	Boliche	C181	1	Clavo	1, 1	na
							Boliche	ni, ni	na
					Ella	1	Clavo	0.996, 1	na
							Boliche	ni, ni	na

CR	2008	Wari	**Baya**	-	Escobon	1	**Baya**	1, 1	na
					**Espartina**)	1	**Baya**	1, 1	1
					**Espino**	1	**Baya**	1, 1	1
					**Estela**	1	**Baya**	1, 1	1
VE	2008	**Jabata II**	Boliche	Clavo	Endrina	1	Boliche	0.997, 1	na
							Clavo	ni, ni	na
					**Esturion**	1[S]	Boliche	1, 1	na
							Clavo	ni, ni	na

Supplementary Table S2. Pairwise relatedness among candidate parents of Iberian lynx. Values are estimates obtained with ML-Relate (Kalinowski et al., 2006) from microsatellite genotypes. Coloured fill indicates values > 0.5 (pink) or in the range 0.25-0.499 (yellow). Please, see Supplementary Table S1 for the sex of each individual.

	Arrayan	Barro	Baya	Boliche	Bonares	Borja	Clavo	Escarlata	Gloria	Iguazu	Jabata2	Maki	Nati2	Nuria	Oscar	Pavon	Rayuela	Roja	Roman	Uda	Viciosa	Wari
Arrayan	1																					
Barro	0.47	1																				
Baya	0	0	1																			
Boliche	0.13	0.1	0	1																		
Bonares	0.37	0.24	0	0	1																	
Borja	0.34	0	0	0.5	0.36	1																
Clavo	0.34	0.48	0	0	0	0.03	1															
Escarlata	0.09	0	0	0.35	0	0.57	0	1														

Gloria	0	0.15	0	0.24	0	0.02	0	0	1													
Iguazu	0	0.2	0	0.22	0.14	0	0	0	0.32	1												
Jabata2	0.58	0.13	0	0	0.09	0.22	0.65	0	0	0	1											
Maki	0.01	0	0	0.03	0.03	0.41	0	0.63	0	0	0	1										
Nati2	0.1	0.13	0	0	0	0.15	0.56	0.05	0	0.09	0.59	0.1	1									
Nuria	0	0	0	0	0.31	0	0	0.06	0	0	0	0.54	0.16	1								
Oscar	0	0.16	0	0.13	0.28	0	0.08	0	0.56	0.25	0.11	0	0	0	1							
Pavon	0.09	0.44	0	0	0	0	0.63	0	0.03	0.3	0.47	0.03	0.63	0	0	1						
Rayuela	0	0	0	0.23	0	0.19	0.06	0.25	0	0	0	0	0.02	0	0	0	1					
Roja	0.03	0.02	0	0.16	0.45	0.5	0	0.19	0	0	0	0.37	0	0.5	0.09	0	0.19	1				
Roman	0	0	0	0.45	0	0.5	0	0.5	0.04	0	0	0.07	0.02	0	0	0	0.63	0.2	1			
Uda	0.04	0.14	0	0.19	0.11	0	0.04	0	0.66	0.17	0.16	0	0	0	0.9	0.05	0	0.05	0	1		
Viciosa	0	0	0	0.24	0	0	0	0.09	0.36	0.5	0.02	0.03	0	0	0.05	0	0.5	0.04	0.53	0.02	1	
Wari	0	0.06	0	0.13	0	0	0	0	0.35	0.5	0.1	0	0	0	0	0.23	0.09	0	0.25	0	0.5	1

References

Kalinowski S.T., Wagner A.P. & Taper M.L. ML-RELATE: a computer program for maximum likelihood estimation of relatedness and relationship. *Molecular Ecology Notes* **6**, 576-579 (2006).
